# Selective determination of 3,5-dihydroxycinnamic acid in urine samples as gluten intake biomarker: high-performance thin-layer chromatography combined with colorimetric detection

**DOI:** 10.1007/s00216-025-05788-1

**Published:** 2025-02-19

**Authors:** A. Martínez-Aviñó, L. Sanjuan-Navarro, Yolanda Moliner-Martínez, M. Roca, C. Ribes-Koninckx, P. Campins-Falco

**Affiliations:** 1 MINTOTA Research Group, Departament de Química Analítica, Facultad de Química, Universitat de Valencia, C/Doctor Moliner 50, 46100 Burjassot, Valencia Spain; 2 Celiac Disease and Digestive Immunopathology Unit, Instituto de Investigación Sanitaria La Fe, 46026 Valencia, Spain; 3 Gastrohepathology Unit, Hospital Universitari i Politècnic La Fe, 46026 Valencia, Spain

**Keywords:** 3,5-DHCA, Target biomarker, Gluten, Colorimetric HPTLC, PDMS-based sensor

## Abstract

The determination of biomarkers is a significant field of analytical chemistry research under continuous evolution that contributes to enhance diagnostics and enable more personalized medicine. Celiac disease is a systemic autoimmune disorder caused by the ingestion of gluten (Glu) proteins found in various cereals. Currently, the only effective way to prevent and manage potential complications is through a strict gluten-free diet (GFD). However, both intentional and unintentional dietary transgressions can occur, often leading to persistent symptoms and ineffective treatment. In this scenario, the development of analytical strategies to detect biomarkers of gluten intake and monitor adherence to a GFD is of significant interest. Herein, we present an analytical strategy based on high-performance thin-layer chromatography (HPTLC) combined with colorimetric detection to estimate 3,5-dihydroxycinnamic acid (3,5-DHCA) as selective biomarker of Glu intake in urine. The approach combined Fast Blue (FB)-doped polydimethylsiloxane (PDMS) membrane with colorimetric HPTLC (RP-C18) giving rise to a selective method to isolate 3,5-DHCA response in urine samples. Detection by visual inspection, image analysis, and spectroscopic response was evaluated and compared. Analytical parameters were estimated showing a good sensitivity (limit of detection (LOD) ≤ 0.8 mg L^−1^) and precision, relative standard deviation (RSD) values < 7%. Analysis of urine samples of celiac patients and control patients was performed, and recovery studies showed satisfactory values (R > 80%). The preliminary results indicated correlation between Glu intake and positive 3,5-DHCA responses. This study demonstrated that FB-doped PDMS membranes-HPTLC is a promising tool for detecting dietary transgressions to the GFD by visual inspection, and subsequent quantitative analysis by image analysis and spectroscopic techniques. Hence, the proposed analytical method contributes to the advance of knowledge about celiac disease, which still remains an important challenge to our society.

**Graphical abstract:**

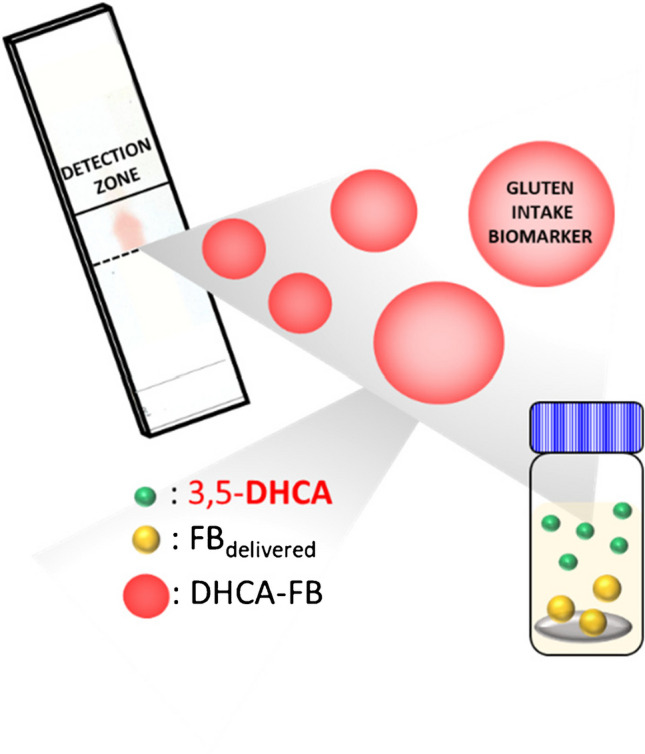

**Supplementary Information:**

The online version contains supplementary material available at 10.1007/s00216-025-05788-1.

## Introduction

The development of analytical methodologies to determine biomarkers for clinical diagnosis has become a very significant area of research toward improved diagnostics, efficient treatments, prevention, and a more personalized medicine. This is particularly relevant when addressing global public health problems, such as celiac disease. Celiac disease (CD) is a systemic autoimmune disorder that is induced by the ingestion of gluten (Glu) protein present in different cereals and in genetically predisposed individuals, even though environmental, infectious, metabolic, and immunological factors are also involved [1, 2].

To date, a strict gluten-free diet (GFD) is the only treatment for CD patients. Nevertheless, compliance with a GFD is not always achieved due to voluntary or involuntary transgressions which can result in persistent symptoms and, hence ineffective treatment and potential co-morbidities such as autoimmune disorders, bone disease (osteopenia/osteoporosis), and chronic anemia [3, 4].

Dietary biomarkers that reflect Glu intake and monitor GFD compliance are of substantial interest in clinical practice although development of analytical methodologies for these compounds are a challenge. This analytical challenge must be addressed from different perspectives to converge into accurate non-invasive biomarkers using sensitive and selective methodologies that allow time and cost-effective dietary follow-up. Remarkably, up to now, GFD adherence monitoring is performed by symptom assessment, dietary questionnaires, intestinal biopsies, serologic tests, and other recent tools as gluten immunogenic peptides [5, 6]. However, all of them show some limitations such as asymptomatic profiles, unreliable dietary interviews, and invasiveness of techniques. In the case of serological tests, failure to detect small or occasional dietary transgressions as well as variability among serological tests has been observed. The use of gluten immunogenic peptides detection in feces or urine samples has demonstrated promising results, but still further characterization and additional research on impact of individual conditions on the results is required [5, 6].

More recently, alkylresorcinols (AR) are under study as biomarkers of Glu intake. The use of ARs in target metabolomics to monitor Glu intake has been addressed from two approaches, determination of the total ARs content and estimation of oxidized ARs metabolites as specific biomarkers. Compounds such as 3,4-dihydroxybenzoic acid (3,4-DHBA), 3-(1,3-dihydroxyphenyl)−1-propionic acid (DHPPA), glycine-DHPPA (Gly-DHPPA), 5-(3,5-dihydroxyphenyl)pvaleentanoic acid (DHPPTA), and 3,5-dihydroxycinnamic acid (3,5-DHCA) have been proposed as potential biomarker of Glu intake from whole grain wheat and rye [7–9]. However, recent reports demonstrated that specificity and precision were improved when detecting DHPPTA and mainly 3,5-DHCA [10]. In addition, their lifetime in urine samples is also higher than the other ARs metabolites, and therefore, they can be used as long-term biomarkers.

Detection and quantification of ARs metabolites in biological samples have been carried out mainly by HPLC [9–12] and gas chromatography (GC) [7, 8, 13] coupled to mass spectrometry (MS), preceded by sample pretreatments to get samples free of interferents, mainly to prevent ionization suppression [14]. The procedures described in these studies have shown satisfactory sensitivity and selectivity. Hollow-fiber liquid-phase microextraction coupled with capillary electrophoresis (CE) has been proposed [15] and ELISA-based methodologies have been also reported [16]. However, achieving analytical responses toward a visual evaluation of GFD compliance remains challenging.

In our previous study, an innovative Fast Blue (FB)-doped polydimethylsiloxane-tetraethyl orthosilicate (PDMS-TEOS) composite has been proposed as a preliminary screening tool to differentiate positive and negative results related with gluten intake using 3,5-DHCA as biomarker. Nevertheless, positive samples still required CapLC coupled DAD detection to isolate and confirm 3,5-DHCA in the samples [17]. Thus, an effort to achieve selectivity but enhancing the analytical performance in terms of direct readouts, portability, cost, and time is still required in order to approximate the implementation to clinical analysis.

In this context, high-performance thin-layer chromatography (HPTLC) is an attractive alternative since it combines the separation efficiency with colorimetric readout. HPTLC can be coupled with MS [18], but alternatively direct colorimetric readouts from the chromatographic layer can be performed, allowing rapid detection and low-cost analysis [19]. However, HPTLC applied to biomarker separation and subsequent detection is still to be explored, since very few studies address this application.

Hence, the objective of this work was to demonstrate the use of colorimetric HPTLC for visual detection and subsequent quantitative analysis of 3,5-DHCA as target biomarker of Glu intake in urine samples to detect alimentary transgressions. For this aim, FB-doped PDMS membranes were used to obtain the colorimetric response. An exhaustive selectivity study was firstly carried out. The analytical performance of HPTLC to estimate the target biomarker under the optimal conditions was performed. In this study, control urines from healthy infants and urines from celiac patients have been used with dual objective, to determine the validity of 3,5-DHCA as Glu biomarker and to estimate the GFD compliance in patients by a visual and colorimetric responses.

## Experimental

### Reagents and materials

FB salt (FB), tetraethyl orthosilicate (TEOS), 3,4-dihydroxycinamic acid (3,4-DHHCA), 3,5-dihydroxycinamic acid (3,5-DHCA), 3,4-dihydroxybenzoic acid (3,4-DHBA), DL-mandelic acid, hippuric acid, vanillic acid, gallic acid, urea, creatinine, and human albumin were purchased from Sigma-Aldrich (Steinheim, Germany). Potassium carbonate and sodium carbonate were obtained from Probus (Badalona, Spain). Potassium chloride and sodium acetate anhydrous were purchased from Panreac (Barcelona, Spain) and sodium chloride and trisodium phosphate dodecahydrate from Manuel Riesgo (Madrid, Spain). Acetic acid was obtained from Scharlau (Barcelona, Spain).

Individual solutions of gallic acid, hippuric acid, vanillic acid, and mandelic acid were prepared in methanol (500 mg L^−1^). Metabolite solutions of 3,5-DHCA, 3,4-DHBA, and 3,4-DHHCA were also prepared in methanol (500 mg L^−1^). Working solution for the target analytes was prepared by the adequate dilution in ultrapure water. Potassium carbonate solution (10%) and acetate buffer 0.1 M (pH = 5) were also prepared.

PDMS elastomer and curing agent (elastomer silicone kit Sylgard 184) were purchased from Dow (USA). Reversed-phase (C_18_) modified silica gel layers (20 × 20 cm) were obtained from Macherey-Nagel (Düren, Germany). Layers had a specific surface of 500 m^2^ g^−1^, 60 Å of pore size, a pore volume of 0.75 mL g^**−**1^ and thickness of 0.15 mm and 2–10 µm of particle size.

### Equipment

UV-vis spectra were recorded using a Cary 60 UV-Vis spectrophotometer equipped with a remote diffuse reflection probe from Harrick Scientific Products (Pleasantville, NY). The diffuse reflection probe has an integral video camera to facilitate selecting the sample spot to be analyzed by providing a visual image. Spectra were recorded in the frequency range of 200–800 cm^−1^. For collection and processing of data, CaryWinUV software from Agilent Technologies was used.

Spectra were also recorded using a Samsung Galaxy A70 Smartphone (Andriod 10) coupled to a GoSpectro portable spectrophotometer equipped with a diffuse reflectance (DR) probe from Goyalab (Bordeaux, France). Halogen lamp of 10 W was used as a light source for spectra registering. Digital images were obtained using a Samsung Galaxy A70 smartphone and GIMP program was used to process images in order to obtain RGB parameters. SX 1200 Power Hairdryer diffuser from Philips (Amsterdam, Netherlands) was used to dry silica gel layers.

### Synthesis of FB-doped PDMS membranes

FB-doped PDMS membranes were prepared by using the previously described procedure in [17]. Briefly, FB derivative reagent was grinded to obtain a fine powder (0.30%); then, PDMS (37%) was added to the reaction beaker and agitated for 5 min until a homogeneous mixture was obtained. Next, TEOS (59%) was added to the previous mixture under continuous stirring. Once a homogeneous mixture was reached, curing reagent (3.7%) was added to the mixture. Finally, membranes were deposited on plastics molds of diameter 1 cm and heated at 40 °C for 24 h. FB-doped PDMS membranes were stored at room temperature in hermetically sealed dark bags remaining stable for a period of 4 months.

### Colorimetric response: HPTLC optimization, separation, and detection

**Fig. 1:**
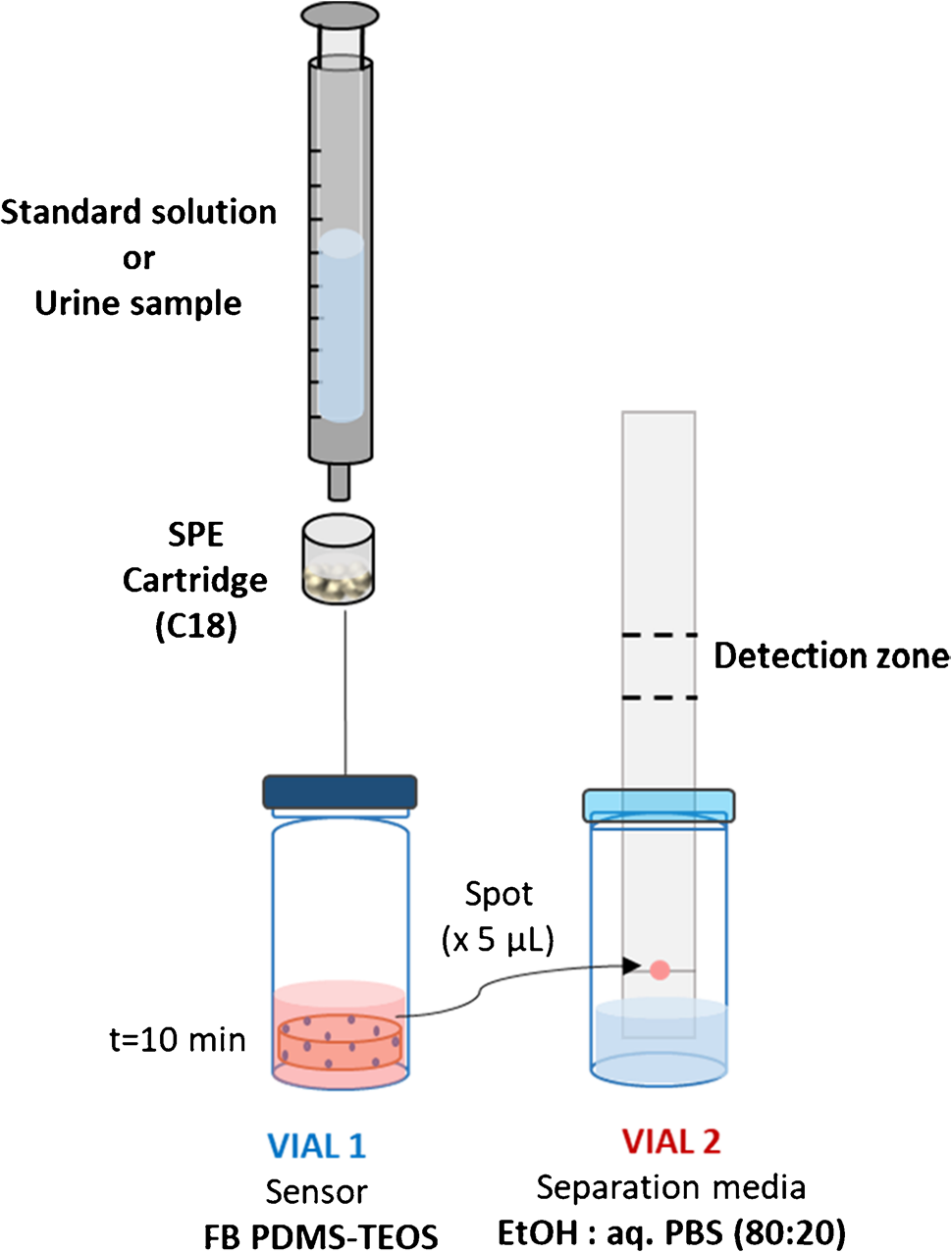
Schematic diagram of the whole analytical procedure and setup proposed for the selective determination of 3,5-DHCA

The colorimetric response was obtained by a simple two-step experimental design. Figure 1 shows the schematic diagram of the whole setup used for the determination of 3,5-DHCA. First, the methanolic SPE extracts (2 mL) of working standard solutions or urine samples were directly added to a vial where the FB-doped PDMS membrane was previously deposited; then, 10 µL of K_2_CO_3_ solution was added. Subsequently, the colorimetric response was obtained after 10 min.

The SPE step was performed by following the procedure described in [17]; C18 silica cartridges (200 mg) were conditioned with methanol (1 mL), water (2 mL), and sodium acetate pH 5.0 (1 mL). Then, 2 mL of working standard solution or urine sample was passed through the SPE cartridge and AR was eluted with methanol (2 mL) directly to the vial where FB-doped PDMS membrane was previously deposited (Vial 1, Fig. 1). Extraction efficiency was evaluated by comparing the response of the methanolic extract (standard solutions, 10 mg L^−1^ 3,5-DHCA, 5 mg L^−1^ gallic acid, and 20 mg L^−1^ 3,4-DHCA) after the SPE procedure with the response of standards, at the same concentration levels, directly prepared in methanol.

Once the derivatization was completed, the 3,5-DHCA isolation and detection were performed following the HPTLC procedure. The methanolic SPE extracts after derivatization with the FB-doped PDMS membranes of standards (gallic acid, hippuric acid, vanillic acid, mandelic acid, and 3,5-DHCA, 3,4-DHBA and 3,4-DHHCA metabolites) and urine samples were deposited with a Camag 10-µL syringe (Hamilton, Switzerland) on 5 × 7 cm HPTLC plates using C_18_ modified silica gel as stationary phase. A volume of 5 µL of each standard solution was placed on a plate. Application step was repeated twice (2 × 5 µL) for high concentration range (up to 20 mg L^−1^) and five times (5 × 5 µL) for low concentration range (up to 4 mg L^−1^) depending on the concentration range in which it is required to work. Separation of the target analytes was performed using a 80:20 ethanol:phosphate buffer solution (PBS) as mobile phase placed in a second vial (Vial 2 in Fig. 1). The plate was developed by ascending chromatography over 6 cm at T_room_. Finally, the colorimetric response (absorbance) was measured at a Rf = 0.5. RGB parameters, as an alternative analytical response, were also obtained from the digital images obtained by the smartphone and processing them with GIMP software. Figure 1 shows the implementation of the HPTLC step in the experimental setup.

### Analysis of urine samples

Urine samples were collected by the Celiac Disease and Digestive Immunopathology Unit at the Instituto de Investigación Sanitaria La Fe. Urine samples from infant with no gluten intake, infant with gluten intake, and patients on GFD were included in this study, after the subsequent informed consent. This study was approved by the Ethics Committee of Instituto de Investigacion Sanitaria La Fe.

Urine samples were analyzed following the procedure described in the “Colorimetric response: HPTLC optimization, separation, and detection” section. Briefly, the methanolic urine extracts after the SPE step were derivatized by using the FB-doped PDMS membrane (Vial 1, Fig. 1). Subsequently, two or five spots (5 µL) of the derivatized solution were placed on the HPTLC plate, and the analytical response was achieved after developing the plate inside of Vial 2 containing the mobile phase (Fig. 1). Finally, the measurement of the response was carried out by using the detection approaches described in the “Colorimetric response: HPTLC optimization, separation, and detection” section. Three different groups of samples were analyzed: negative control group formed by infant with no gluten intake, positive control group formed by infant with gluten intake, and, finally, the patient group composed of four CD patients on GFD. Celiac disease patients were on a GFD for more than 1 year. Since no modifications of the diet were recommended nor before nor on the sampling days, samples were collected at day 1 and day 3, in order to detect potential transgressions. Hence, two sets of samples were analyzed; first set of samples was collected at day 1 (P_i_−1), and after three days (P_i_−3).

Additionally, accuracy and matrix effects were assessed through the measurement of spiked urine samples with known amount of the target analyte. For this aim, urine samples spiked with 3,5-DHCA (10 mg L^−1^) were analyzed following the proposed method. These spiked samples were processed by the whole analytical method, and the results were compared with the values achieved with 3,5-DHCA standard solutions processed under the same conditions.

## Results and discussion

### DHCA as gluten biomarker

In a previous study, it was reported that FB-doped PDMS membranes directly added to a methanolic solution of 3,5-DHCA induced FB release, and formation of the azoderivative compound. The colorimetric response in methanolic extracts of urine samples could be used as a negative/positive screening tool correlated with Glu intake. The morphology of FB-doped PDMS membranes was studied in [17]. Using this assay, it was demonstrated that FB specifically stains compounds with resorcinol ring of 3,5-DHCA; however, several compounds with reactive phenolic hydroxyl functional group could result in positive responses in the absence of 3,5-DHCA. Thus, positive responses required a confirmatory study by means of a chromatographic separation to isolate 3,5-DHCA from potential interfering compounds. So, a deeper study toward a better understanding of the FB-doped PDMS selectivity is required. In the preliminary experiments, compounds such as gallic, vanillic, hippuric, caffeic, mandelic, and chlorogenic acids were tested. In addition, other metabolites such as 3,4-DHHCA and 3,4-DHBA were also studied. Figure S1A shows the spectra obtained for the target biomarker and the tested compounds, compared to the blank signal.

Nitrogen of the FB diazonium group salt is retained in coupling with the reactive activating group of the phenolic group. The interaction mechanism is currently well understood [20]. Coupling mostly occurs in the *para* position to the phenolic activating group, unless the position is already occupied, then the substitution takes place in the *ortho* position. As can be seen from the molecular structure of 3,5-DHCA, its resorcinol ring presents two-hydroxyl activating group with non-occupied *para* positions, which facilitates the reaction with FB reagent providing high analytical responses (see Fig. S1A). On the other hand, 3,4-DHHCA, 3,4-DHBA, only present one activating group in *para* or *ortho* position, providing a low analytical response, compared to 3,5-DHCA.

The two-hydroxyl activating group with non-occupied *para* positions of gallic acid also induced a significant response for these compounds; however, there was a band shift to lower wavelengths compared to 3,5-DHCA. Indeed, the wavelength was explained by the different colors of the derivative formed (see Fig. S1B). Caffeic acid, vanillic acid, and chlorogenic acid showed responses at concentrations higher than 80 mg L^−1^, which was in accordance with their structure. Mandelic acid and hippuric acid were also tested but no analytical response was observed as their molecular structure does not present activating groups in their resorcinol ring [21].

From a quantitative perspective, sensitivity (estimated as the slope of the calibration graphs [22]) for gallic acid was (6.6 ± 0.5)·10^−2^ L mg^−1^ in the working concentration range of 0.4–5 mg L^−1^. Meanwhile, the sensitivity for 3,4-DHHCA and 3,4-DHBA (2–40 mg L^−1^) was (2.6 ± 0.1)·10^−3^ and (2.10 ± 0.07)·10^−3^ L mg^−1^, respectively. Remarkably, the target biomarker provided a sensitivity value of (18.0 ± 0.4)·10^−2^ L mg^−1^. Despite the sensitivity differences, the presence of matrix components needs to be considered, and separated from the 3,5-DHCA signal, as significant variation in the slope can occur when other reactive compounds were present in the solution. Experimentally, the slopes of the calibration curves were statistically not comparable in mixtures containing the target biomarker, and the other compounds. As expected, in this study, the variation in the slope depended mainly on the composition of the mixture. On the other hand, the kinetic behavior of the potential target biomarker compared with the studied interfering compounds differed (see Fig. S1C). The increase of the signal with time was mainly depending on 3,5-DHCA, since constant responses were obtained for phenolic compound, DHHBA, and 3,4-DHBA. Taking into account these results together with the high inter-individual variability in urine samples, HPTLC separation was evaluated in order to demonstrate the viability of 3,5-DHCA as biomarker of Glu intake with an operational-friendly device.

### HPTLC separation and detection

In this work, the hypothesis, and hence the novelty, was that colorimetric HPTLC combined the 3,5-DHCA visual detection, separation, and quantification in one single step. In the preliminary studies, different stationary phases were tested in order to achieve the optimum signal response not only in terms of intensity but also in terms of uniformity of the obtained spots. Nanosilica gel (NanoSiO_2_), reversed-phase C_18_ (RP-C_18_), NH_2_ modified nano silica gel and CN modified nano silica gel plates were studied as supports for the chromatographic analysis of 3,5-DHCA. As observed in Fig. S2A, the use of NanoSiO^2^ and RP-C18 as stationary phases resulted in a more intense signal. NH_2_ modified nano silica gel produced low color intensity spot, while poor coloration was obtained when CN modified nano silica gel plates were tested. As expected, interaction between RP-C18 and the azocompound formed was favored providing a satisfactory separation efficiency and low signal dispersion; therefore, RP-C_18_ was the selected stationary phase as obtained color spots were more consistent allowing to perform reliable measurements.

Different mobile phases were evaluated for 3,5-DHCA isolation; volumes of 5μL were applied five times per analyte with the aim of ensuring a suitable sensitivity and selectivity for the tested compounds. Water, PBS, EtOH, acetone, and acetonitrile were tested. Mobile phases composed of acetone and water provided poor analyte separations obtaining insufficient retention factor, mainly due to the low eluent strength. Hexane:H_2_O was also evaluated as mobile phase obtaining efficient separation; nevertheless, it was ruled out as hexane is not considered an ecofriendly solvent.

In this sense, following the trends of green analytical chemistry, different compositions of acetonitrile:H_2_O and EtOH:H_2_O were tested, obtaining in both cases a good analyte separation. According to Byrne et al., acetonitrile and EtOH are considered a recommended ecofriendly solvent in some green solvent selection guides, making both suitable mobile phases [23]. However, EtOH:H_2_O provided a more uniform coloration and a higher analytical response making it an optimal mobile phase for this case of study. Rf values for 3,5-DHCA and the interfering species were then studied. Different compositions of EtOH:H_2_O were evaluated in order to improve 3,5-DHCA isolation as can be seen in Fig. S2B. It was seen that higher proportion of EtOH caused an increase of the eluting strength obtaining high Rf values. Conversely, by increasing the proportion of water, a decrease of Rf values was observed; this decrease was particularly evident for 3,5-DHCA, thus allowing for a better isolation of the analyte of interest. However, too high proportions of water also resulted in long time analysis and vague analytical responses. Taking this into consideration, a proportion of 80:20 (EtOH:H_2_O) was chosen to perform this work. Some inorganic acids and bases as well as buffer solutions have been used as modifiers in chromatography in order to control the pH of mobile phases and to get sharper peaks. Implementing this concept to HPTLC, carbonate buffer solution (pH = 11.2), PBS (pH = 8.1), and citrate buffer solution (pH = 5.5) were evaluated. Aqueous PBS was proven to provide the most well-defined and uniformly colored spots when used as the aqueous phase. According to the results observed, 80:20 (EtOH:PBS) was selected as the optimal mobile phase for further experimentation in this work.

**Fig. 2:**
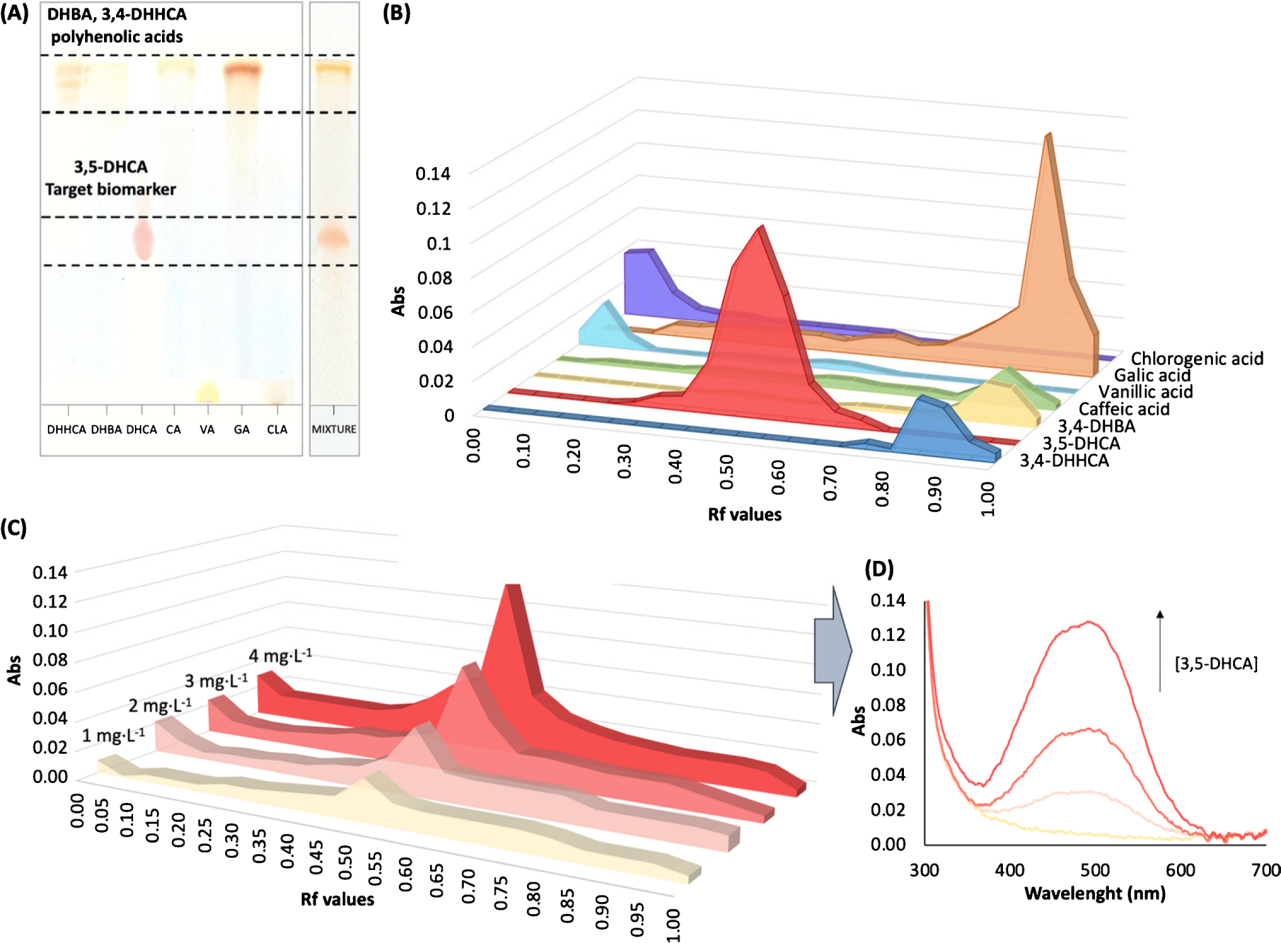
**A** C18 chromatographic layer for the different analytes and their mixture after the separation. **B** Chromatogram Abs vs Rf values calculated at the optimum condition. **C** Variation of the chromatograms as a function of the concentration of 3,5-DHCA (1–4 mg L^−1^). **D** UV-vis spectra obtained with the DR spectrometer for each spot of 3,5-DHCA at different concentrations (1 mg L^−1^, 2 mg L^−1^, 3 mg L^−1^, and 4 mg L^−1^)

Figure 2A shows the HPTLC plate after the analysis; as can be observed, the visual inspection of the plate allowed the identification of the target analyte isolated from the rest of the studied compounds, both when compared with individual standards and in mixtures. As shown, selective visual detection of 3,5-DHCA was demonstrated taking into account the chromatographic resolution in the Rf chromatogram (see Fig. 2B). The spot corresponding to 3,5-DHCA was at Rf = 0.5; meanwhile, 3,4-DHHCA and 3,4-DHBA eluted at higher Rf, like gallic acid. However, vanillic acid and chlorogenic acids provided low Rf values.

In this work, detection was performed by using two different readouts in order to evaluate the possibilities of the proposed biomarker system: DR and imagine analysis. DR was performed by a conventional laboratory instrument and by a reflectance probe connected to a smartphone. Digital analysis was carried out by RGB analysis of the chromatographic film images. Figure 2C shows the chromatograms obtained by measuring DR with the reflectance probe connected to the smartphone. As expected, there was a satisfactory correlation between absorbance and concentration. The spectra obtained for each spot at different concentrations demonstrated an increase in absorbance as a function of concentration, with a maximum at 520 nm (see Fig. 2D). It should be noted that the spectra in Fig. 2D were measured by using five spots of the standard solutions. These results corroborated the potential use of the proposed methodology at low 3,5-DHCA concentration level.

The application of the procedure required a SPE step (see the “Experimental” section), and hence, the extraction efficiencies (EE, %) for the target analyte and interfering compounds were evaluated. These values were 89 ± 4%, 71 ± 7%, and 60 ± 9% for 3,5-DHCA, gallic acid, and 3,4-DHHCA, respectively. The results indicated that after the SPE step, not only the target analyte was found in the methanolic extract, but also other FB-derivatizable phenolic compounds, such as gallic acid or 3,4-DHHCA highlighting the need for the isolation/separation step to estimate the 3,5-DHCA as target analyte.

### Analytical parameters

The use of 3,5-DHCA as biomarker in biological samples to evaluate the gluten intake is complex, since the content can vary according to the intake, individual patient factors and sampling conditions among others. The figures of merit of the proposed strategy were determined by using aqueous standards, and processed under the experimental conditions described in the “Colorimetric response: HPTLC optimization, separation, and detection” section (Fig. 1). Urine samples were analyzed under the same conditions. Quantitative analysis was carried out using a DR spectrometer. Additionally, and in order to endow portability to the proposed methodology, a smartphone-spectrometer coupled to a fiber optic probe and image analysis was also evaluated as detection systems. DR measurements were performed taking into account a Rf = 0.5 and digital images were obtained scanning chromatographic layers using a smartphone; corresponding RGB chromatograms were performed by using the GIMP free program. Chromatograms were obtained for each color coordinate (red, green, blue) at different Rf values. Best result was provided by blue (B) color component, in terms of precision and linearity.

The figures of merits of the 3,5-DHCA determination in HPTLC plates are shown in Table 1. The analyte quantification was carried out by using different instrumentations following the optimized procedure at a wavelength of 500 nm. The limits of detection (LOD) and limits of quantification (LOQ) were calculated as 3s_blank_/*b* and 10s_blank_/*b*, respectively, where S_blank_ is the standard deviation of the blank and *b* is the slope of the regression line. Linearity range, sensitivity, and precision of the methods were evaluated.

**Table 1:** Figures of merit by using DR spectrometer and smartphone DR-probe, and smartphone-digital image analysis

Instrument	Intercept (a ± s_a_)	Slope (b ± s_b_) (mg^−1^ L)	*R*^2^	Linearity range (mg L^−1^)		LODs (mg L^−1^)	LQDs (mg L^−1^)
DR spectrometer	0.005 ± 0.002	(7.40 ± 0.02) 10^−2^	0.998		2.6–20	0.80	2.60
Smartphone-DR spectrometer probe	0.007 ± 0.003	(1.59 ± 0.02) ·10^−3^	0.999		3.3–20	1.00	3.30
Smartphone-imagen analysis (RGB-B component)	0.007 ± 0.004	(1.68 ± 0.03) ·10^−3^	0.998		3.6–20	1.10	3.60

Results achieved were highly satisfactory as can be seen in Table 1. Good correlations and appropriated sensitivity expressed as LOD and LOQ values were obtained for both working concentration levels. The content of 3,5-DHCA in urine samples can differ from one patient to another; consequently, the results observed ensure a quality analysis at a wide range of concentrations. Precision was evaluated by using the relative standard deviation (RSD, %), achieving RSD values < 2% for the laboratory DR spectrometer and RSD < 7% for portable instrumentation. The figures of merit by using portable instrumentation were similar to those obtained by using the traditional laboratory DR spectrometer; hence, portable instrumentation has been proved to be a suitable alternative.

Concentration ranges can be variable in urine sample due to the high inter-individual variability; thus, in order to assess the quantitative performance of the proposed method, a low concentration range (1 to 4 mg L^−1^) was also registered. It should be noted that, given the novelty of this compound as potential biomarkers and the current state of the art in their study as biomarkers correlated with gluten intake, no limit values are yet available, and hence, likewise Glu immunopeptides and serological biomarkers, detection limits are determined by the detection limits of the analytical method employed. These concentration levels were reached by preconcentrating the sample in five spots (5 µL). Under these conditions, it was demonstrated that using the DR spectrometer, LOD of 0.25 mg L^−1^ and LOQ of 0.8 mg L^−1^ could be achieved, linearity was *R*^2^ = 0.997 and precision, as RSD value, was 4%. Hence, the sensitivity of the proposed methodology can be modulated as a function of the expected 3,5-DHCA concentration level in the urine sample and the requirements of the analysis by defining the number of spots on the HPTLC plate.

Additionally, the global assessment of the proposed methodology was evaluated by using the HEXAGON tool, in which sample treatment, separation/detection technique, and method characteristics noticeably play a key role in defining the figures of merit of the analytical procedure [24]. The assessment showed that the proposed method is sustainable, particularly due to its low carbon footprint, low residue production, cost-effectiveness, and improvement of the portability [25]. The HEXAGON results (Fig. S3) reflected low penalty points assigned to these criteria compared to other methodologies, such as HPLC, employed to analyze gluten intake using 3,5-DHCA as biomarker [17].

On the other hand, Table 2 summarizes previously described analytical methods proposed to determine AR and their metabolites in biological samples. In these approaches, most determination methods relied on instrumentation such as HPLC or GC mainly combined with MS detection to achieve satisfactory selectivity and low detection limits. Unlike the previous studies, in the described method, the approach was based on the simplification of the instrumentation to develop an affordable and selective analytical strategy that allowed a visual positive/negative test and subsequent simple colorimetric detection of 3,5-DHCA as indicator of the GFD compliance. Evidently, the simplification of the instrumentation entailed a decrease on the sensitivity. Indeed, the LODs achieved by the proposed method are higher than those achieved by HPLC-MS or GC-MS. However, the proposed methodology has proven to differentiate between negative and positive control urine samples which indicated adequate sensitivity for this application (see the “Application to urine samples” section). In addition, preconcentration of the target analyte would be still possible if the practical application would require a lower LOD. The aforementioned simplification was also accompanied by a decrease on the analysis time; as can be seen in Table 2, the analysis time for the proposed approach was 12 min, but it should be remarked that a multisample analysis can be performed by using multiport devices, particularly, in this work ten samples were analyzed simultaneously. Considering these features, along with the methodology and analytical parameters described in this work, the proposed approach can be regarded as a green and sustainable analytical strategy to estimate 3,5-DHCA in urine samples.

**Table 2:** Analytical methods previously described for the determination of AR and their metabolites in biological samples

Analyte	Sample	Sample treatment	Separation technique	Detection	LOD	Analysis time per sample (min)	Ref
AR C17-C25	Plasma	-	UPLC-QTOF	MS	—	—	[26]
AR C17-C25	Plasma	Derivatization	GC	MS	—	35	[27]
AR C17-C26	Serum	SPE	LC	MS	138 μg⋅g^−1^	20	[12]
DHPPA	Plasma	Hydrolysis centrifugation	LC	MS	—	25	[28]
3,4-DHBA/DHPPA	Urine	LLE centrifugation	LC	MS	1 ng⋅mL^−1^ (LOQ)	17	[29]
					0.5 ng⋅mL^−1^ (LOQ)		
3,5-DHCA/3,4-DHBA/DHPPA	Urine	—	GC	MS	0.1 μmol⋅L^−1^ 0.3 μmol⋅L^−1^ 0.2 μmol⋅L^−1^	35	[28]
3,5-DHCA	Urine	SPE FB-doped PDMS	Miniaturized LC	DAD	60 μg⋅L^−1^	20	[17]
3,5-DHCA	Urine	SPE FB-doped PDMS	HPTLC	Visual inspection Image analysis	0.25 mg⋅L^−1^ 0.80 mg⋅L^−1^	12* (10 samples)	This work

*Use of a 10-sample multiport device

### Application to urine samples

**Fig. 3:**
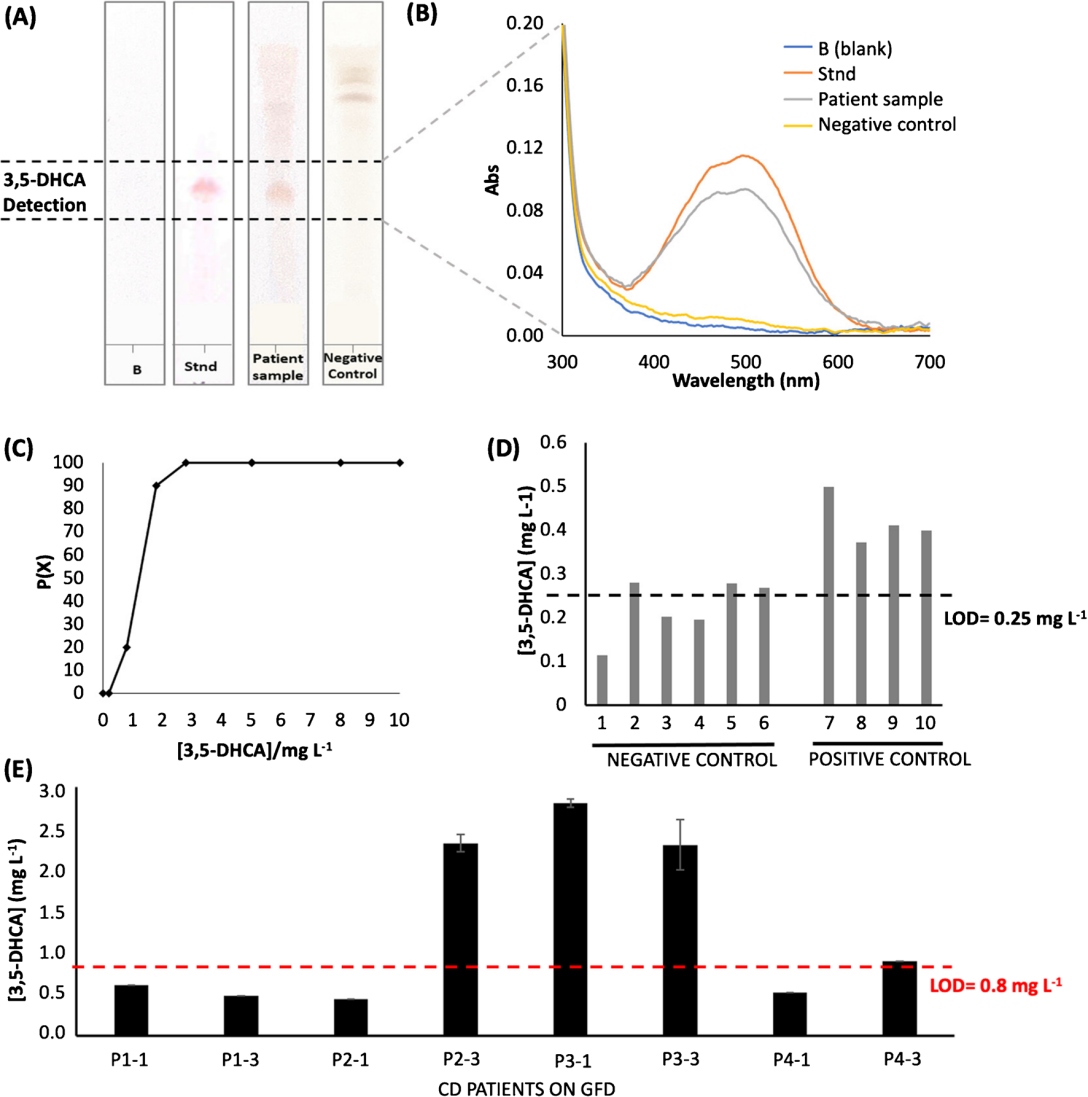
**A** Comparison of the chromatographic layer for a blank, 3,5-DHCA standard, a CD patient sample, and a negative control. **B** Spectra obtained for the chromatographic layer. **C** P(X) of positive responses as a function of the 3,5-DHCA concentration. **D** Concentration estimated in control samples (positive and negative). **E** Analysis of CD patient samples

The proposed methodology has been applied for the analysis of urine samples. In this study, urine samples for celiac patients and control urines (healthy infants) were used to demonstrate the potential use of 3,5-DHCA as a biomarker correlated with Glu intake. Negative control samples, positive control samples, and samples from CD patients were analyzed. Figure 3A shows the comparison of the HPTLC plates for a blank, a 3,5-DHCA standard, a negative control sample, and a CD patient sample. These results, together with the spectra in Fig. 3B, revealed that CD patients provided an analytical response that corresponded to the 3,5-DHCA response. At that Rf, no signal was observed for the negative control sample. This was corroborated with the UV-vis spectra, where only a band at 520 nm was observed for the 3,5-DHCA standard, and for the CD patient sample, that would mean a potential gluten transgression.

Matrix effects were evaluated by using spiked urine samples with 3,5-DHCA following the procedure described in the “Analysis of urine samples” section, and comparing the analytical response with a 3,5-DHCA standard processed under same experimental conditions [30]. The recovery values were in the range 80 ± 5% for all the tested samples, indicating that the matrix components did not significantly interfere with the analyte detection and quantification. Satisfactory accuracy was achieved with relative errors below 20%.

In order to study the reliability of the proposed system, a qualitative characterization in urine samples was performed by evaluating false positives, since the proposed device can be considered a yes/no test by visual inspection of the chromatographic layer. The probability of having positive responses (P(x)) as a function of the 3,5-DHCA concentration was calculated. It should be remarked that control samples were used to ensure the low concentration of the biomarker and the LOD was selected as cutoff value. As can be seen in Fig. 3C, the unreliability range was between 0.8 and 1.8 mg L^−1^ (at 95% as probability level). Remarkably, this interval was quite narrow even selecting the cutoff concentration at the detection limit. In addition, the probability of having false negatives at concentration higher than 1.8 mg L^−1^ was only 10%. It should be remarked that the use of lower concentration range (by increasing the number of sample spots), resulted in comparable results in terms of qualitative reliability.

Negative control sample and positive control samples were subsequently compared. Figure 3D compares the responses obtained. It should be noted that this experiment was performed by using six sample spots (LOD = 0.25 mg L^−1^) taking into account the low 3,5-DHCA content. As can be seen, gluten intake (positive control group) gave rise to a higher response in the 3,5-DHCA detection zone of the HPTLC plate compared to the negative control group. Indeed, negative control samples were below the detection limit in all cases, which is in agreement with previous results.

Finally, four CD patient samples on GFD were analyzed. Samples were taken in two different days—First samples were taken (P_i_−1), and after 3 days, the second samples were collected for each patient (P_i_−3). These samples corresponded to adolescents (10–14 years old) and the working concentration interval used in this case was up to 20 mg L^−1^. Figure 3E shows the results obtained for these groups of samples. As can be seen, 3,5-DHCA concentration was higher than in control samples. It is important to remark that the control samples pertain to very young infants and urine composition may be naturally different from older children and adolescents.

Samples P1-1, P1-3, P2-1, P4-1, and P4-3 were below or at the detection limit (LOD = 0.8 mg L^−1^). It should be noted, that these patients were not strictly compliant with the diet. Indeed, the results were variable depending on the urine sample and in addition occult gluten intake cannot be discarded. The content of 3,5-DHCA in samples P2-3, P3-1, and P3-3 was higher than the LOQ and could be quantified. The results for these samples were in agreement with previous studies where dietary transgressions were suspected by clinicians, due to slightly positive CD serological markers.

Table 3 summarizes the results obtained for the tested samples in terms of positive and negative responses and the values for positive samples if they could be quantified. The results indicated that alimentary transgression can be correlated with the positive response of 3–5-DHCA; however, there was individual variability for the concentration level. These results suggested that the measurement of 3.5-DHCA celiac patient on GFD can be an indicator of alimentary transgression, and the use of the proposed methodology can be a potential tool to detect it. Future works will be necessary to advance in the knowledge, in particular of different celiac patients’ population.

**Table 3:** Qualitative and quantitative study of urine samples from celiac patients

Patient	Sample	Response (P(X))	[3,5-DHCA] (mg L^−1^)
Patient 1	P1-1_-SDAY1_	Negative (< 5%)*	< LOD
	P1-3-_SDAY3_	Negative (< 5%)*	< LOD
Patient 2	P2-1-_SDAY1_	Negative (< 5%)*	< LOD
	P2-3-_SDAY3_	Positive (< 5%)**	2.32 ± 0.12
Patient 3	P3-1-_SDAY1_	Positive (< 5%)**	2.80 ± 0.04
	P3-3-_SDAY3_	Positive (< 5%)**	2.30 ± 0.30
Patient 4	P4-1-_SDAY1_	Negative (< 5%)*	< LOD
	P-4–3-_SDAY3_	Positive (75%)*	< LOD

*P(X) false negatives; **P(x) false positives

## Conclusions

In summary, an analytical tool based colorimetric HPTLC combined with FB-doped PDMS membrane has been developed to estimate 3,5-DHCA as selective biomarker of Glu intake in urine samples. The combined strategy advances the development of colorimetric tests to evaluate alimentary transgressions of CD patients on GFD. The proposed methodology is based on the colorimetric response obtained for 3,5-DHCA with FB-based PDMS membrane, and subsequent separation on RP-C18 chromatographic layer. The results indicated satisfactory analytical parameters in terms of precision, selectivity, and sensitivity. Furthermore, sensitivity of the proposed test can be modulated as a function of the number of spots, depending on the requirements of the samples. Control samples and CD patient samples corroborated the potential use of the proposed strategy, demonstrating the correlation of the HPTLC chromatographic signal with the gluten intake. The reliability as yes/no test was also demonstrated, showing a quite narrow unreliability interval even selecting the cutoff concentration at the detection limit. Finally, the results obtained for positive samples, together with the comparison with serological studies, suggested that the level of 3.5-DHCA in urine samples of celiac patient on GFD can be an indicator of alimentary transgression, and the use of the proposed methodology can be a potential tool to detect it. Besides, portable systems have been demonstrated to read out the response obtained, which provided portability/sustainability to the analytical device. Future works are still necessary to advance the knowledge of these diseases.

## Supplementary Information

Below is the link to the electronic supplementary material.

Supplementary file1 (DOCX 933 KB)
