# Supplementary material for: Selective determination of 3,5-dihydroxycinnamic acid in urine samples as gluten intake biomarker: high-performance thin-layer chromatography combined with colorimetric detection
Source: Anal Bioanal Chem. 2025 Feb 19;417(10):2061–72. doi: 10.1007/s00216-025-05788-1 (PMC11961461; doi:10.1007/s00216-025-05788-1)
Supplement: Supplementary file 1 — Supplementary file1 (DOCX 933 KB) [file 216_2025_5788_MOESM1_ESM.docx]

**Supporting information**

**Selective determination of 3,5- dihydroxycinnamic acid in urine samples as gluten intake biomarker: high performance thin layer chromatography combined with colorimetric detection**

1. Martínez-Aviñó^1^, L. Sanjuan-Navarro^1^, Y. Moliner-Martínez^1^*, M. Roca^2^, C. Ribes-Koninckx^2^, P. Campins-Falco^1^

^1^MINTOTA Research Group, Departament de Química Analítica, Facultad de Química, Universitat de Valencia.

C/Doctor Moliner 50, E46100- Burjassot, Valencia. Spain.

^2^Celiac Disease and Digestive Immunopathology Unit, Instituto de Investigación Sanitaria La Fe, 46026, Valencia, Spain.

Gastrohepathology Unit, Hospital Universitari i Politècnic La Fe, 46026, Valencia, Spain.

**3. RESULTS AND DISCUSSION**

**3.1. 3,5-DHCA as gluten biomarker**


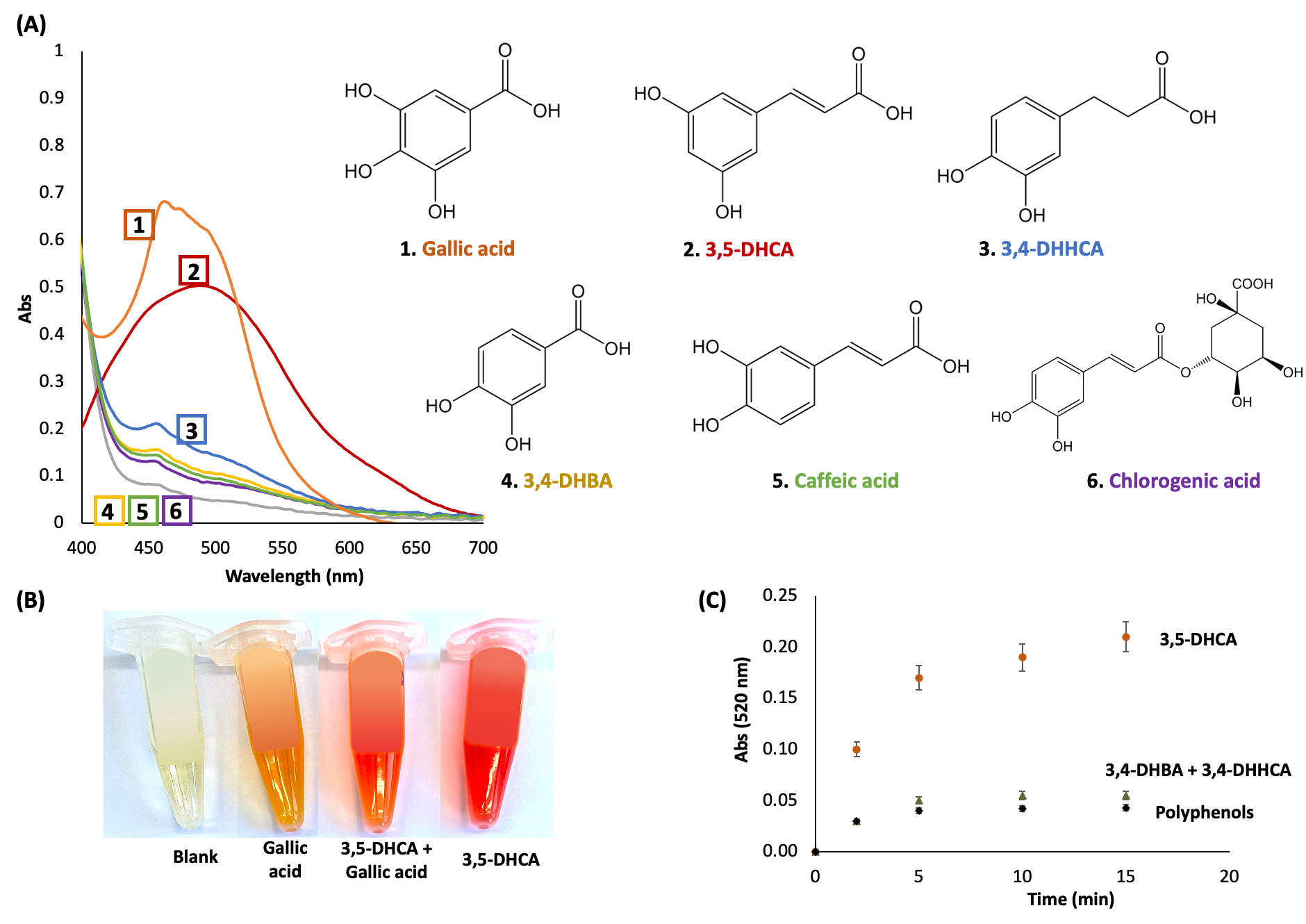


**Figure S1.** A) UV-vis spectra for the target biomarker, 3,5-DHCA and for the studied compounds: 3,4-DHBA, 3,4-DHHCA, Gallic acid, Caffeic acid and chlorogenic acid; B) Image of the derivatized compounds compared with the blank; C) Variation of the response over time for 3,5-DHCA, a mixture of 3,4-DHBA and 3,4-DHHCA and for a mixture of polyphenols at a concentration of 20 mg·L^-1^.

**3.2. HPTLC separation and detection**

**
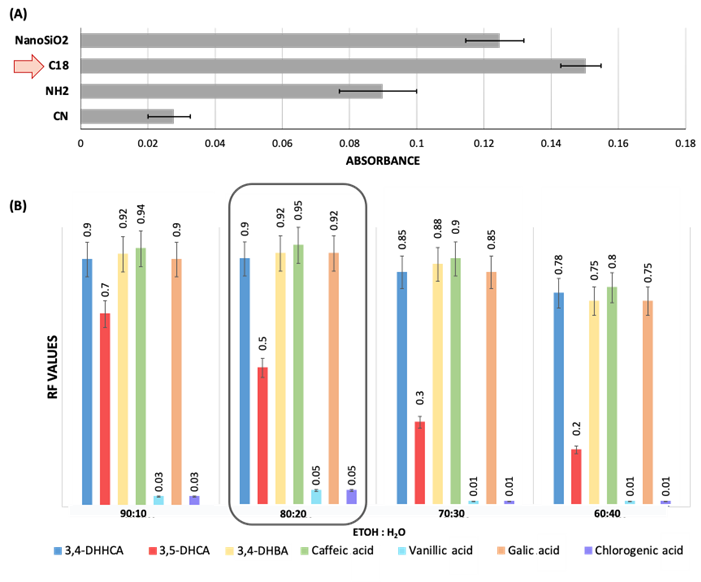
**

**Figure S2.** A) Registered values of color intensity for different stationary phases (NanoSiO_2_, RP-C_18_, NH_2_ modified NanoSiO_2_ and CN modified NanoSiO_2_) for 3,5-DHCA at a concentration of 20 mg·L^-1^; B) Variation of Rf Values for different compositions of EtOH:H_2_O as mobile phase using RP-C_18_ as stationary phase. Each experiment was performed in triplicate.

**3.3. Analytical parameters**


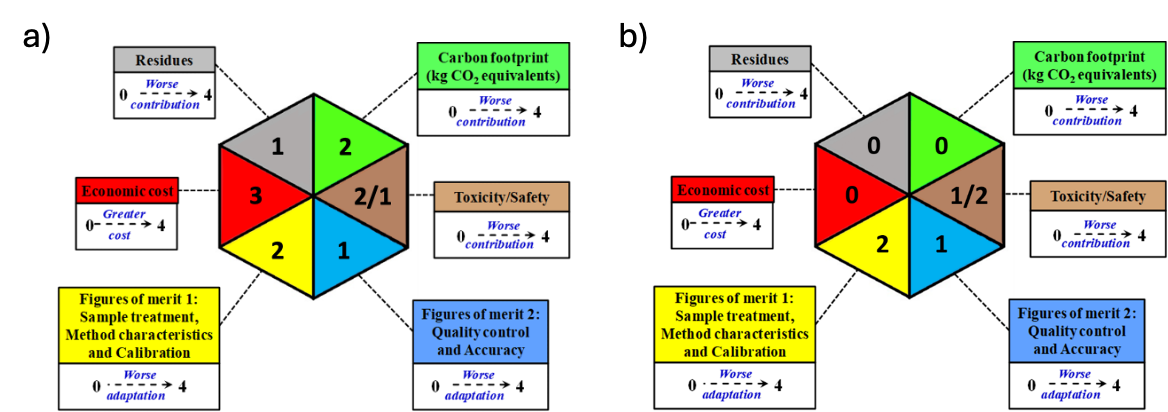


**Figure S3.** Regular hexagon pictogram for the evaluation of 3,5- dihydroxycinnamic acid as target biomarker of gluten intake by a) in-tube SPME-Capillary LC-DAD and b) colorimetric HPTLC.
